# Photooxidation crosslinking to recover residual stress in decellularized blood vessel

**DOI:** 10.1093/rb/rbaa058

**Published:** 2021-03-13

**Authors:** Jintao Wang, Lingwen Kong, Alidha Gafur, Xiaobo Peng, Natalia Kristi, Jing Xu, Xingshuang Ma, Nan Wang, Rose Humphry, Colm Durkan, Haijun Zhang, Zhiyi Ye, Guixue Wang

**Affiliations:** 1 Key Laboratory of Biorheological Science and Technology (Chongqing University), Ministry of Education, State and Local Joint Engineering Laboratory for Vascular Implants, Bioengineering College of Chongqing University, Chongqing 400030, China; 2 Department of Cardiothoracic Surgery, Central Hospital of Chongqing University, Chongqing Emergency Medical Center, Chongqing 400014, China; 3 The Nanoscience Centre, University of Cambridge, Cambridge CB3 0FF, UK; 4 National Local Joint Engineering Laboratory for Biomedical Material Modification, Dezhou, Shandong 251100, China

**Keywords:** residual stress, opening angle, mechanical property, decellularized artery, photooxidation crosslinking, finite element method

## Abstract

Decellularization method based on trypsin-digestion is widely used to construct small diameter vascular grafts. However, this method will reduce the opening angle of the blood vessel and result in the reduction of residual stress. Residual stress reduced has an adverse effect on the compliance and permeability of small diameter vascular grafts. To improve the situation, acellular blood vessels were treated with glutaraldehyde and photooxidation crosslinking respectively, and the changes of opening angle, circumferential residual strain of native blood vessels, decellularized arteries and crosslinked blood vessels were measured by means of histological examination, scanning electron microscopy (SEM) and transmission electron microscopy (TEM) in this study. The opening angle of decellularized arteries significantly restored after photooxidation crosslinking (*P* = 0.0216), while that of glutaraldehyde crosslinking blood vessels reduced. The elastic fibers inside the blood vessels became densely rearranged after photooxidation crosslinking. The results of finite element simulation showed that the residual stress increased with the increase of opening angle. In this study, we found at the first time that photooxidation crosslinking method could significantly increase the residual stress of decellularized vessels, which provides biomechanical support for the development of new biomaterials of vascular grafts.

## Introduction

The products of blood vessel transplantation have been widely used in the clinic [1–3]. However, small-caliber artificial blood vessels have been associated with thrombosis, aneurysm, poor compliance, and low patency rates exist in small-caliber artificial blood vessels, which is problematic for artificial blood vessel research. Vasoconstriction is unable to occur according to the pulsation of blood when the artificial vessel wall is in contact with blood [4].

This effect can result in the formation of stress concentration, which in turn can encourage platelet aggregation and thrombosis. This causes vascular stenosis that decreases the patency rate. Mechanical properties strongly influence the patency rates of small-caliber blood vessels [5]. Technological advances in tissue engineering have resulted in a new type of artificial blood vessel that was created based on decellularized extracellular matrix (ECM) technology to promote the recovery of physiological functions [6, 7]. Preparation methods for decellularized vessels include physical, chemical, enzymatic digestion and composite methods [8–11]. Different methods and processes of decellularization will change the structural and mechanical properties of ECM [11].

At present, the most effective decellularization method is to combine physical, chemical and enzyme applications [12–17]. Trypsin can separate the cells from structural proteins and completely eliminate the cells, which is widely used in various research and preparation of decellularized products. There are many studies on decellularization of arterial and muscle tissues using trypsin. In addition to preserving the composition and structure of the tissue, the decellularized vascular graft must be able to withstand the pulsating pressure generated by the blood flow and be comparable to the compliance of the native artery. The trypsin-based decellularization schemes destroy the elastic and collagen fibrous tissue in the artery, which reduce the ductility and opening angle and cause a reduction in the residual stress of the vessel [18, 19]. The reduction of residual stress by acellular method based on trypsin caused adverse effect on mechanical behavior including supporting blood pressure and preventing stress concentration.

The residual stress is a critical factor in maintaining the normal physiological function and physiological state of the arterial tissue. The residual stress can prevent the stress concentration and maintain the compliance of the vessel. A reduction of residual stress caused by the decellularization method led to problems such as low permeability of the vascular graft after long-term use [20]. The zero-stress state is the most basic state for performing mechanical analysis and studying vascular reconstruction. In order to understand the structural and functional properties of the vessel wall, analyzing the stress-growth relationship from the zero-stress state of the vessel wall is necessary. The opening angle highlights residual stresses existing in the arterial wall and characterizes the extent of opening of the vascular ring with a radial cut [2, 21]. Therefore, the zero-stress state of the vessel wall and opening angle has received significant attention and research [22–25]. However, previous studies have demonstrated that decellularization caused the decrease of opening angles, which indicated that residual stress decreased with decellularization [21].

Arterial tissue becomes soft due to the removal of cells, especially blood vessel and organ transplantation could not meet the mechanical requirements of clinical application. Therefore, researchers have improved the mechanical properties of acellular scaffolds through various crosslinking schemes (physical methods, chemical crosslinking agents, natural cross-linking agents) [26–28]. Physical methods include photooxidation crosslinking and thermal dehydrogenation, and chemical crosslinkers mainly include epoxy compounds, glutaraldehyde. Glutaraldehyde-crosslinked materials have been applied to preserve tissue in cardiovascular surgery. However, calcification, toxicity and mechanical failure are the disadvantages of glutaraldehyde-crosslinked materials [29–31]. Methylene blue-mediated photooxidation crosslinking was applied in construction of tissue engineering scaffold. Methylene blue-mediated photooxidation crosslinking has many obvious advantages, such as preventing thrombosis and increasing mechanical tensile strength [32–34]. Although it is encouraging that crosslinking can improve the mechanical properties of decellularized materials, the reduction of residual stress has a greater impact on the decellularized materials. However, no studies have explored the effect of crosslinking methods on residual stress in decellularized vessels.

The aim of this present study was to reconstruct the residual stress of decellularized vessels in using different crosslinking methods. The variation of opening angles was observed in order to determine the changes in residual stress. We confirmed that the opening angles of decellularized vessels were smaller than that of native arteries; the opening angles were increased through photooxidation crosslinking. Histology, transmission electron microscopy (TEM) and scanning electron microscopy (SEM) were used to confirm the removal of cells and compare the microstructure of decellularized vessels and crosslinked vessels. Differences were observed and compared among native arteries, decellularized arteries and crosslinked arteries by measurement of opening angles and residual strains. The change of residual stress under different opening angles was simulated by finite element method.

## Materials and methods

### Harvest and preparation of rat carotid artery

Healthy adult male Sprague-Dawley (SD) rats (*n* = 30, 300 ± 25 g) were purchased from the Animal Experimental Center of Third Military Medical University (China). All animal experiments were performed according to the protocols that were approved by the Ethical Committee of Chongqing University. Native vessels were isolated from the neck of the experimental SD rats. Rats were anesthetized by intraperitoneal injection of 7% chloral hydrate solution (0.9 ml/100g) and euthanized by injecting 3–5 ml of air into the tail vein. The right common artery was quickly obtained and immediately immersed in phosphate-buffered saline (PBS) solution. After removing impurities from the vessels, the vessels were stored at 4°C.

The common carotid artery samples were subdivided equally into four test groups, which were (i) the native arteries (*n* = 5), (ii) the decellularized arteries (*n* = 5), (iii) the decellularized arteries with glutaraldehyde crosslinking (*n* = 5) and (iv) the decellularized arteries with photooxidation crosslinking under different conditions (*n* = 15).

### Decellularization of carotid arteries

The arterial samples were immersed in 4 ml of distilled water and shaken for one day (speed 120 r/min, temperature 37°C). The arterial samples were then immersed in PBS and separately processed by freezing treatment (at a temperature of −80°C) and water bath (at a temperature of 37°C) for three cycles of 2 h each. The arteries were treated with 70% ethanol solution at room temperature for 1 day, during which the ethanol solution was changed every 2 h. Finally, the arterial samples were treated with 0.125% trypsin in PBS and placed on the shaking table for 1.5 h.

### Glutaraldehyde crosslinking

After decellularization, five decellularized arteries obtained were used for glutaraldehyde crosslinking (G-crosslinked). Glutaraldehyde with initial concentration of 500 mg/ml (50%) was diluted to a concentration of 2.5 mg/ml (0.25%) by PBS. Diluted glutaraldehyde was poured into decellularized arteries until the lumen was full. Then decellularized arteries were crosslinked by soaking in glutaraldehyde with a concentration of 2.5 mg/ml for 30 min at 4°C. After crosslinking with glutaraldehyde, the arteries’ lumens were washed with PBS, and arteries immersed in PBS were shaken for 6 h. During this, the solution was changed every hour.

### Photooxidation crosslinking under different conditions

After decellularization, 15 decellularized arteries obtained were used for photooxidation crosslinking under different conditions. Based on the previous study, the concentration was set at 0.01%, and the UV exposure time was 2 h [35]. To explore the effects of different concentrations and exposure time on opening angles in decellularized vessels, we set three different combinations of concentrations and exposure time for photooxidation crosslinking. The crosslinking solution was composed of methylene blue and hydrogen peroxide; the concentrations of methylene blue used were 0.01%, 0.03% and 0.005%. Oxygen was fed into the fresh crosslinking solution at a rate of 1.5 ml/min for 30 min in dark conditions. Decellularized arteries were soaked in the various concentrations of crosslinking solution after the aeration. Then the crosslinking reaction was driven by medium wavelength ultraviolet light. For each of the concentrations of crosslinking solutions, the arteries were exposed to UV for different durations: 0.01% for 2 h, 0.03% for 4 h and 0.005% for 1 h. Arteries were then washed in PBS solution three times and stored at 4°C until subsequent testing was performed.

### Histological examination

Histological analyses were conducted on the samples from native and decellularized arteries. Both native and decellularized samples were cut into sections of 5 μm and fixed in 4% paraformaldehyde over 24 h. Next, the samples were washed in distilled water, dehydrated in graded ethanol, paraffin-embedded, and sectioned into 4 μm thicknesses. The samples were stained with H&E to highlight nuclear materials, Masson’s trichrome to highlight collagen and Meta-diphenol. Photooxidation crosslinking samples were stained with Masson’s trichrome and Meta-diphenol.

### Measurement of residual strain and opening angles

**Figure 1.**
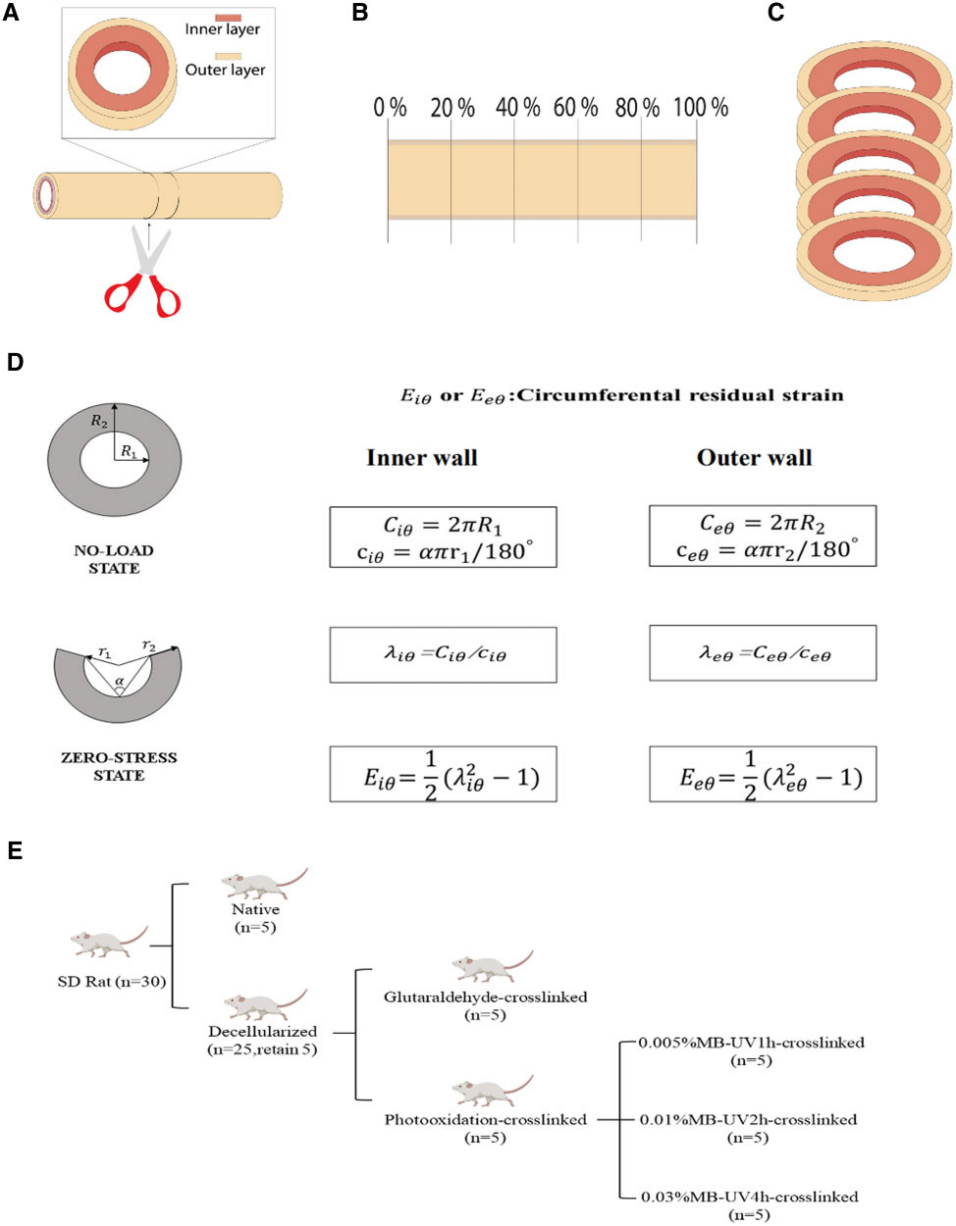
Cutting the blood vessel along the radial direction (**A**). The vessels were segmented according to the position ratio (**B**). The whole blood vessel gives five rings (**C**). Principle and method of measuring opening angles and residual strain (**D**). Experimental scheme design and experimental group presentation (**E**)

Eighteen carotid arteries were used for measurement of opening angles and residual strain. The opening angles were measured directly to reveal the change in residual stress of the vessels [36]. Both native and decellularized arteries were poured into PBS and divided into vascular rings 5 mm in length from the proximal end to distal end (Fig. 1A). The axial lengths were measured for each vascular ring, and the mean of axial length of both sides was defined as the axial length of each vascular ring. The total length of the entire vessel was defined as the sum of all the axial lengths of the vascular rings. The location-allocation of each ring was the ratio of length (from the proximal end to ring) and the total length of the whole vessel (Fig. 1B and C).

The lateral face and cross-section of vascular ring were photographed by stereomicroscope. The vascular ring was cut along the axial direction of the blood vessel and left to stand for 30 min. The face of the cut was subsequently photographed by stereomicroscopes. The opening angle (∝) was defined as the angle formed between two lines extending from the tips of the opened sample to the midpoint of the inner arc of the sample. The circumferential residual strain of the vessel was divided into the circumferential strain of the inner wall ${(E}_{i\theta })$ and the circumferential strain of the outer wall ($E_{e\theta }$).

Based on the assumption that the blood vessels are continuous media, orthotropic and volume incompressible, according to the strain tensor definition of Green, the strain of the blood vessel can be obtained [37]. The inner wall circumferential strain and outer wall circumferential strain can be defined as follows:

$$E_{i\theta }=\frac{1}{2}(\lambda_{i\theta }^{2}-1)\;\;\;\;\;\;\;\;\;\;\;\;\;\;\;\;\;\;E_{e\theta }=\frac{1}{2}(\lambda_{e\theta }^{2}-1)$$

Here, $\lambda_{i\theta }$ represents inner wall circumferential elongation ratio,$\;\lambda_{e\theta }$ represents outer wall circumferential elongation ratio.

$$\lambda_{i\theta }=C_{i\theta }/c_{i\theta }\;\;{\;\;\;\;\;\;\;\;\;\;\;\;\;\;\;\;\;\lambda }_{e\theta }=C_{e\theta }/c_{e\theta }$$

$C_{i\theta }$ and $C_{e\theta }$ represent the perimeter of inner wall and outer wall at no-load state respectively,$\;c_{i\theta }$ and$\;c_{e\theta }$ represent the perimeter of inner wall and outer wall at zero-stress state respectively.

### Scanning electron microscopy

The vessels were placed in a 4°C refrigerator and fixed with 2.5% glutaraldehyde for one day. The vessels were then dehydrated through an ethanol gradient, critical point dried, sputter-coated with gold and finally observed using SEM.

### Transmission electron microscopy

The vessels, pre-fixed with 3% glutaraldehyde, were fixed with 1% osmium tetroxide and subsequently dehydrated in a series of acetone. The dehydrated vessels were passed through a dehydrating agent and an epoxy resin (model Epon812) permeate, then embedded. The vessels were prepared into an ultrathin slice with a thickness of 50 nm by ultramicrotome, stained with uranyl acetate and lead citrate, and examined with H600 IV.

### Finite element simulation of residual stress

We used the method of re-closing the opening angle under a certain stretch ratio to obtain the residual stress inside the vessel wall. The finite element software ABAQUS (Dassult Systems 2016) was used to simulate the closure of zero-stress blood vessels with opening angles of 35°, 70°and 98°, respectively. Using the knowledge from previous studies on the inner and outer walls of blood vessels, the length of the arterial wall blood vessel model was set to 5 mm, the thickness of the intima and adventitia was set to 0.2 mm long, and the inner diameter was 1 mm. The material density was set at 1150 kg/m3 and Poisson’s ratio was 0.45. Arterial models were meshed with reduced eight-node brick elements (C3D8R).

The carotid arterial wall was assumed to be nonlinear, isotropic and incompressible. The material has been defined by the Mooney–Rivlin constitutive equation. The strain energy function is given by $W=C_{10}\left(I_{1}-3\right)+C_{01}(I_{2}-3)$, where $C_{10}$ and $C_{01}$ represent material constants; $I_{1}$ and $I_{2}$ represent the first and second strain invariants.

Arterial tissue of the wall material model was determined by curve-fitting data from uniaxial tensile tests of carotid arterial tissue [38]. The values used in this study are provided in Table 1. A reference point was established on the road surface to define the coupling constraints between the load surface and the reference point. Fixed restraint was applied to the other end of the model so that the load surface movement coincides with the restraint surface.

**Table 1.** The mechanical properties of artery

Material constants	$\;C_{10}$(MPa)	$C_{01}$
	2.101	−2.091

### Statistical analysis

All experimental data are expressed as mean ± standard deviation (SD). All calculations were performed using Graph Pad Prism version 7.0 (Graph Pad Prism Software, Inc., San Diego). A student’s *t*-test was performed to the determine the statistical differences between two groups (mean ± SD), while a one-way analysis of variance was performed for over three groups (mean ± SD), followed by Tukey’s post-hoc test to conduct statistical significance. *P*-values < 0.05 indicated statistical significance. *P* values < 0.01 indicated highly significant.

## Results

### Decellularization caused fiber’s separation and cell removal

**Figure 2.**
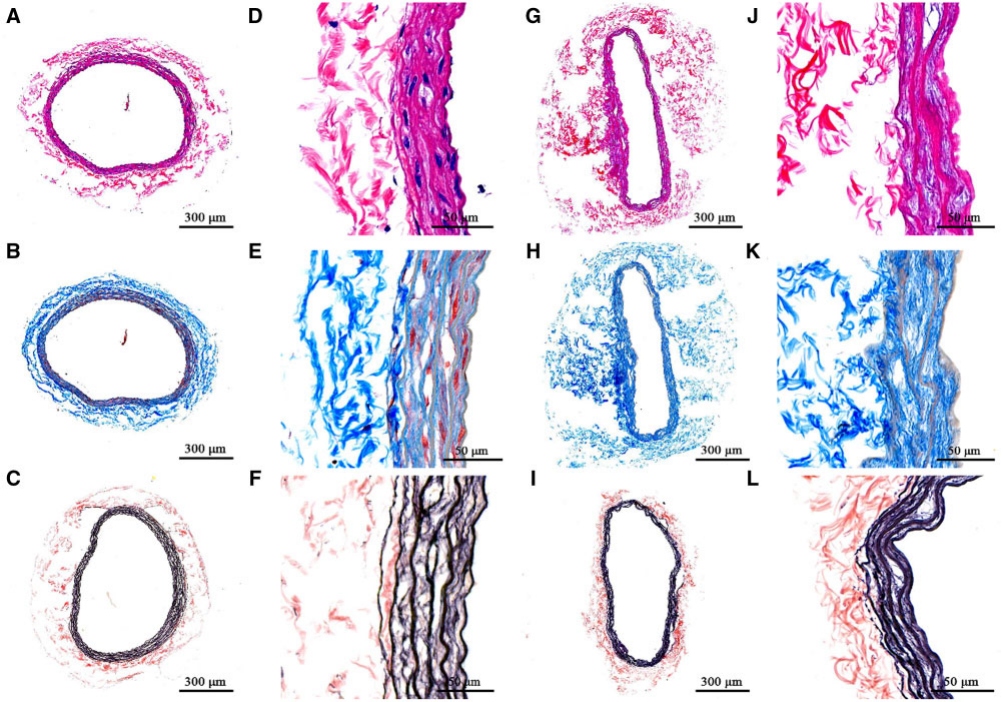
Histological results of native and decellularized. HE staining results of arteries before (**A** and **B**) and after decellularization (**G** and **J**). Masson staining results of arteries before (**B** and **E**) and after decellularization (**H** and **K**). Diphenol staining results of arteries before (**C** and **F**) and after decellularization (**I** and **L**). The scale bars in the images were 300 µm

Two carotid arteries were used for histological examination. The vessel walls of native arteries were divided into dense inner structure and loose outer structure, where a large number of cells existed in the inner structure, especially (Fig. 2A and D). The outer structure of decellularized arteries becomes loose, and the presence of nuclear material was not observed in decellularized arteries (Fig. 2G and J). As the results of Mason’s dyeing show, the cytoplasm and fibers were stained red in the inner structure, and the outer structure was mainly formed of a network structure composed of collagen fibers (Fig. 2B and E). After decellularization, decellularized arteries’ inner structure contained no evidence of the cytoplasm and fibers (Fig. 2H). The collagen fibers’ network became loose through the destruction of the interactions between collagen fibers and other components during the decellularization process (Fig. 2K). After the treatment of Meta-diphenol dyeing, the inner structure of native arteries was stained black due to the high density of elastic fiber, present in a state of layer distribution, and the outer structure of native arteries was stained reddish-brown with collagen fibers. However, elastic fibers from the inner structure of decellularized arteries became loose, resulting in separation between elastic fiber layers. The separation between the inner and outer structures revealed that the intravascular elastic fiber network was destroyed through the decellularization process.

### Comparison of opening angles and residual strain between native and decellularized arteries

**Figure 3.**
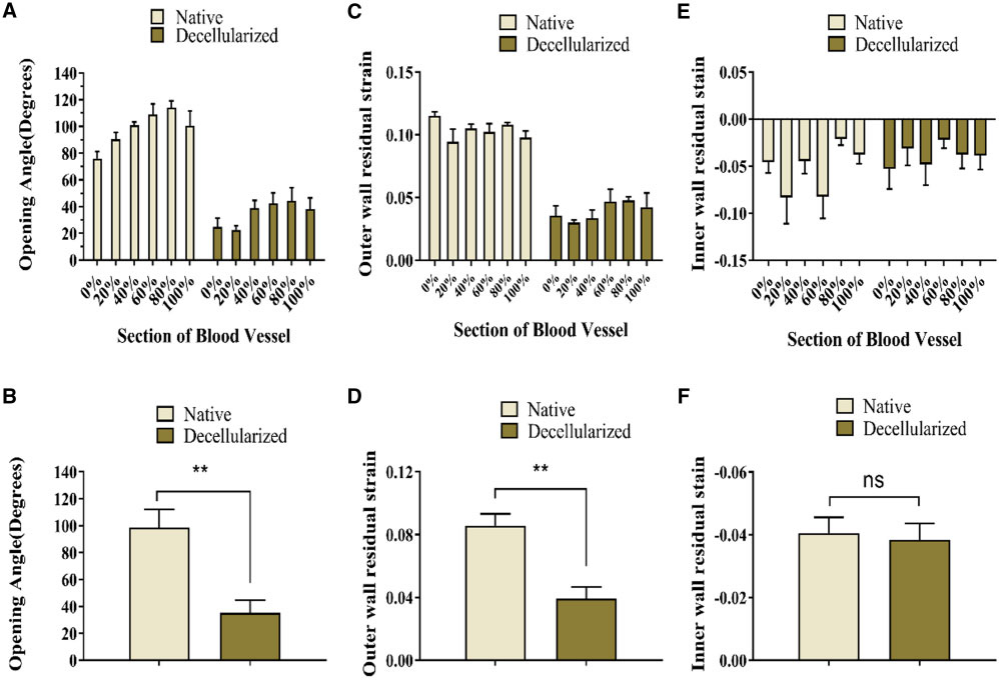
Comparison of the biomechanical properties of decellularized arteries and native arteries. (**A**) Opening angles were significantly greater in native arteries compared with decellularized arteries. (**C**) The outer wall residual strain decreased significantly after decellularization. (**E**) The inner wall experienced little change in residual strain. (**B**), (**D**) and (**F**) revealed whether there was a significant difference in opening angles, outer and inner wall residual strain between decellularized arteries and native arteries, respectively. *Corresponds to a *P* < 0.05; **Corresponds to a *P* < 0.01; ns, not significant

The opening angles of native arteries were higher than those of decellularized arteries (Fig. 3A and B). The mean opening angles was 98.48°± 13.65 for native arteries and 35.21°± 9.24 for decellularized arteries (Fig. 3B). Outer wall residual strain decreased after decellularization (Fig. 3B and D). Since the inner wall of both the native and decellularized arteries were under pressure, the associated residual strain was negative. The decellularized arteries’ inner wall residual strain barely changed and had no significant difference with that of native arteries (Fig. 3E and F).

### Select the best crosslinking method to restore residual stress

**Figure 4.**
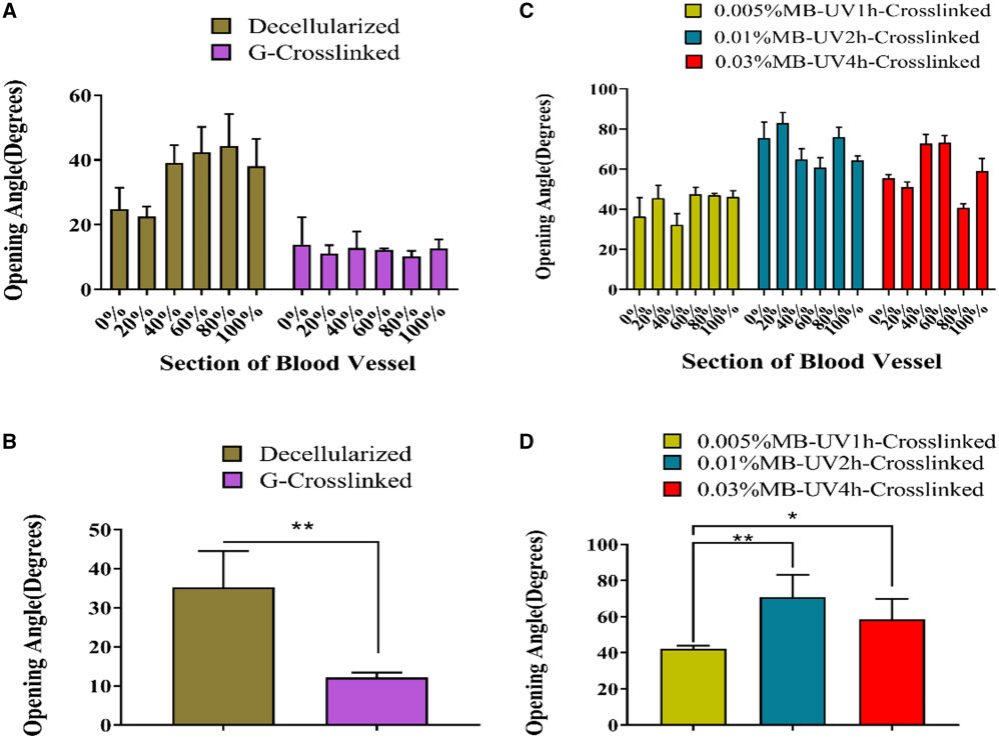
Comparison of opening angles of decellularized arteries with glutaraldehyde crosslinking (G-crosslinked) or photooxidation crosslinking under different conditions (0.005% MB-UV1h-crosslinked, 0.01%MB-UV2h-crosslinked, 0.03%MB-UV4h-crosslinked). (**A**) Opening angles of G-crosslinked arteries were smaller than that of decellularized arteries. (**B**) There was a significant difference between G-crosslinked arteries and decellularized arteries. (**C**) Comparison of the opening angles of decellularized arteries with photooxidation crosslinking under different conditions. (**D**) There is a significant difference among opening angles of decellularized arteries with photooxidation crosslinking under different conditions. *Corresponds to a *P* < 0.05, **Corresponds to a *P* < 0.01

The opening angles of G-crosslinked arteries were less than those of decellularized arteries at every segment (value counting mean ± SD: G-crosslinked artery: 12.15°± 1.30; decellularized artery: 35.21°± 9.24) (Fig. 4A and B). All three photooxidation methods increased the opening angles compared with those of decellularized arteries, but to a varying extent. The increase of the opening angles of 0.01%MB-UV2h-crosslinked arteries was more significant than that of 0.03%MB-UV4h-crosslinked arteries or the 0.005%MB-UV1h-crosslinked arteries at every segment (Fig. 4D).

### 0.01% Mb-UV2h-crosslinked arteries biomechanical test

**Figure 5.**
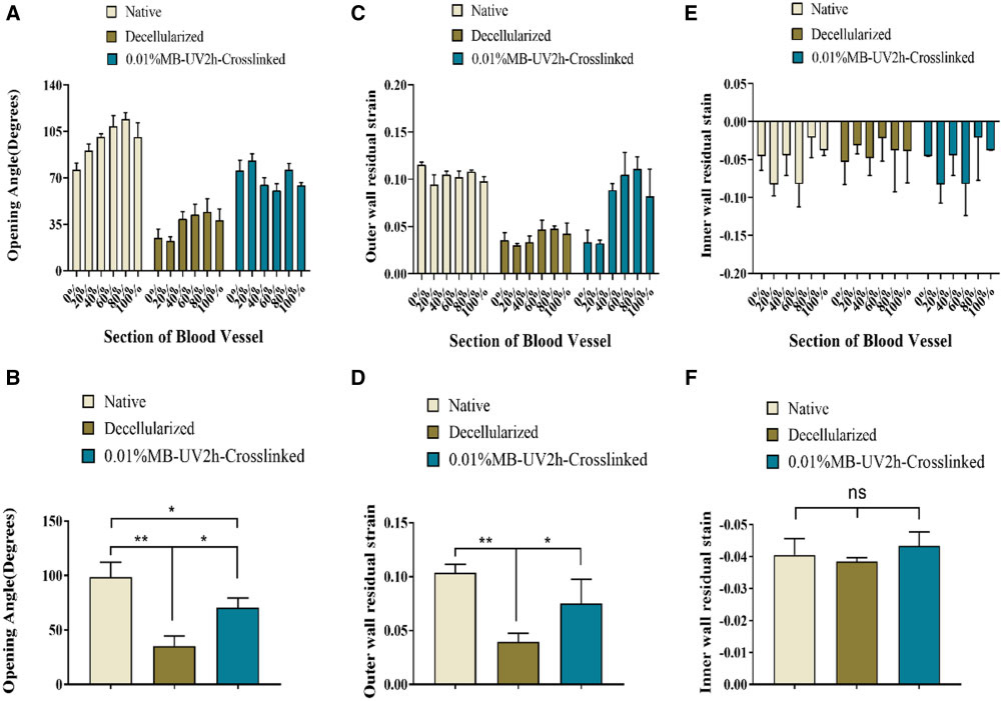
Comparison of biomechanical parameters among native, decellularized and 0.01% MB-UV2h-crosslinked arteries. (**A**) Opening angles of crosslinked arteries appeared to increase compared with decellularized arteries. (**C**) The outer wall residual strains increased though crosslinking. (**E**) The residual strain on the inner wall did not appear to change significantly. (**B**), (**D**) and (**F**) reveal whether opening angles, outer wall residual strains and inner wall residual strains have a significant difference among native, decellularized and 0.01% MB-UV2h-crosslinked arteries.*Corresponds to a *P* < 0.05; **Corresponds to a *P* < 0.01; ns, not significant

After photooxidation crosslinking treatment, the opening angle of each segment of decellularized vessel was found to increase. However, the increase gradually decreased from the near heart to the far end (Fig. 5A). The mean value of the opening angles for 0.01%MB-UV2h-crosslinked arteries was 70.67°, which was much larger than that of the decellularized arteries (Fig. 5B). The outer wall residual strain of the 0.01%MB-UV2h-crosslinked arteries did not differ from the decellularized vessels in the first 20% of the segments. However, the outer wall residual strains of the 40–100% segment were much larger than those of the decellularized vessels (Fig. 5C). The change of the outer wall residual strain trend was consistent with the change of opening angles (Fig. 5B and D). The inner residual strains of the 0.01%MB-UV2h-crosslinked arteries did not change compared with the decellularized vessels (Fig. 5E). The difference in the inner residual strains was not significant among any of the decellularized arteries, the 0.01%MB-UV2h-crosslinked arteries, or native arteries (Fig. 5F).

### Scanning electron microscope

**Figure 6.**
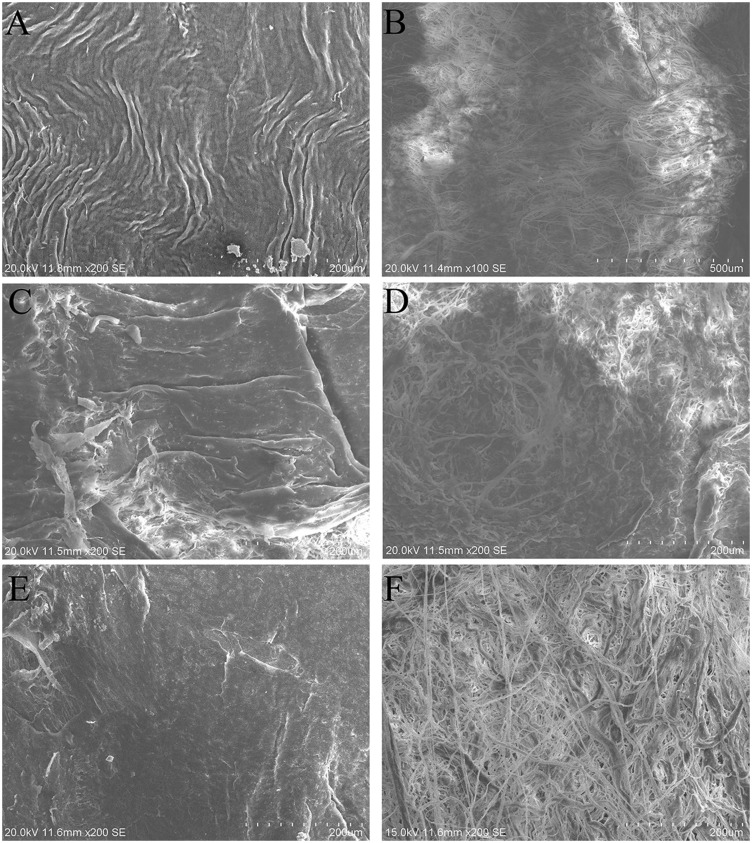
SEM of intimal and adventitial surfaces of native, decellularized and 0.01%MB-UV2h-crosslinked arteries. (**A**) and (**B**) represent the intima and adventitial surface of the native arteries;(**C**) and (**D**) represent the intima and adventitial surface of the decellularized arteries; (**E**) and (**F**) represent the intima and adventitial surface of the 0.01%MB-UV2h-crosslinked arteries

The intima surface of native arteries showed damascene endothelial layers (Fig. 6A). Fibers were revealed and extracellular matrix (ECM) displayed in the intima surface of decellularized and 0.01%MB-UV2h-crosslinked arteries (Fig. 6C and E). Dense bundles of rippled collagen fibers were revealed in the adventitia surface of the native arteries (Fig. 6B). However, a network of collagen fibers with shallow pores became loose in the adventitia surface of the decellularized arteries (Fig. 6D). It was observed that for the 0.01%MB-UV2h-crosslinked arteries, the collagen fibers became denser than those of decellularized arteries, suggesting fibers were re-connected and extended by photooxidation crosslinking (Fig. 6F).

### Transmission electron microscope

**Figure 7.**
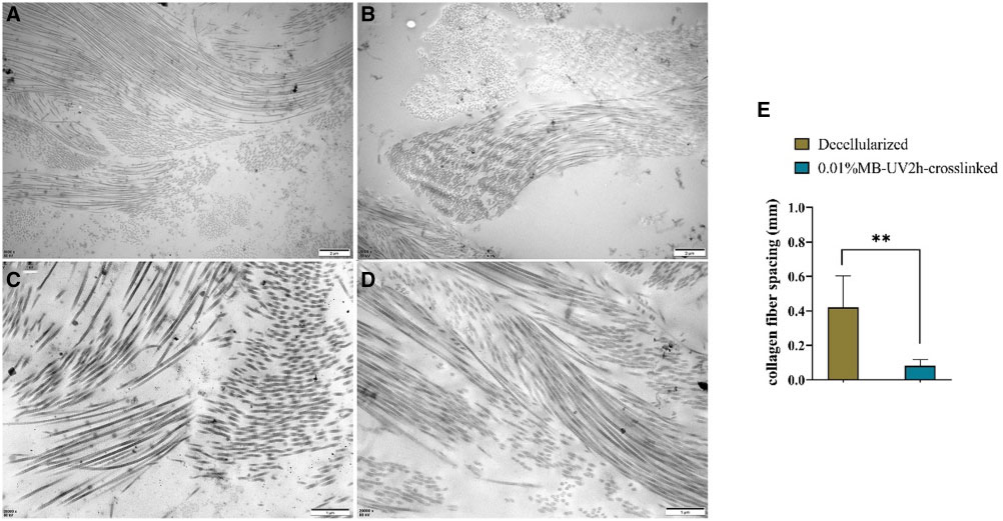
TEM of decellularized and 0.01%MB-UV2h-crosslinked arteries. Example images are shown for (**A**) decellularized and (**B**) 0.01%MB-UV2h-crosslinked arteries. Zoomed-in images of (**C**) decellularized and (**E**) 0.01%MB-UV2h-crosslinked arteries showed details of collagen fibers. The comparison of spacing of collagen fibers between decellularized and 0.01%MB-UV2h-crosslinked arteries (**E**)

TEM revealed both the collagen fiber density and the spacing of connections between fibers, in both the decellularized and 0.01%MB-UV2h-crosslinked arteries. 0.01%MB-UV2h-crosslinked arteries had denser regions of collagen fibers than in decellularized arteries (Fig. 7A and B). The average spacing of collagen fiber was counted and calculated by software Image J by selecting five pairs of collagen fibers in TEM images of decellularized and 0.01%MB-UV2h-crosslinked arteries (Fig. 7C and D). The average spacing of collagen fibers in 0.01%MB-UV2h-crosslinked arteries was smaller significantly than that in decellularized arteries (Fig. 7E). In the 0.01%MB-UV2h-crosslinked arteries, the collagen fibers appeared to be closely contacted with bunches (Fig. 7D). However, in the decellularized arteries, the spacing among collagen fibers appeared to have increased (Fig. 7C). Collagen fibers were arranged very neatly along the longitudinal direction in 0.01%MB-UV2h-crosslinked arteries. However, in decellularized arteries, collagen fibers were arranged chaotically and divided into many short segments.

### Finite element simulation

**Figure 8.**
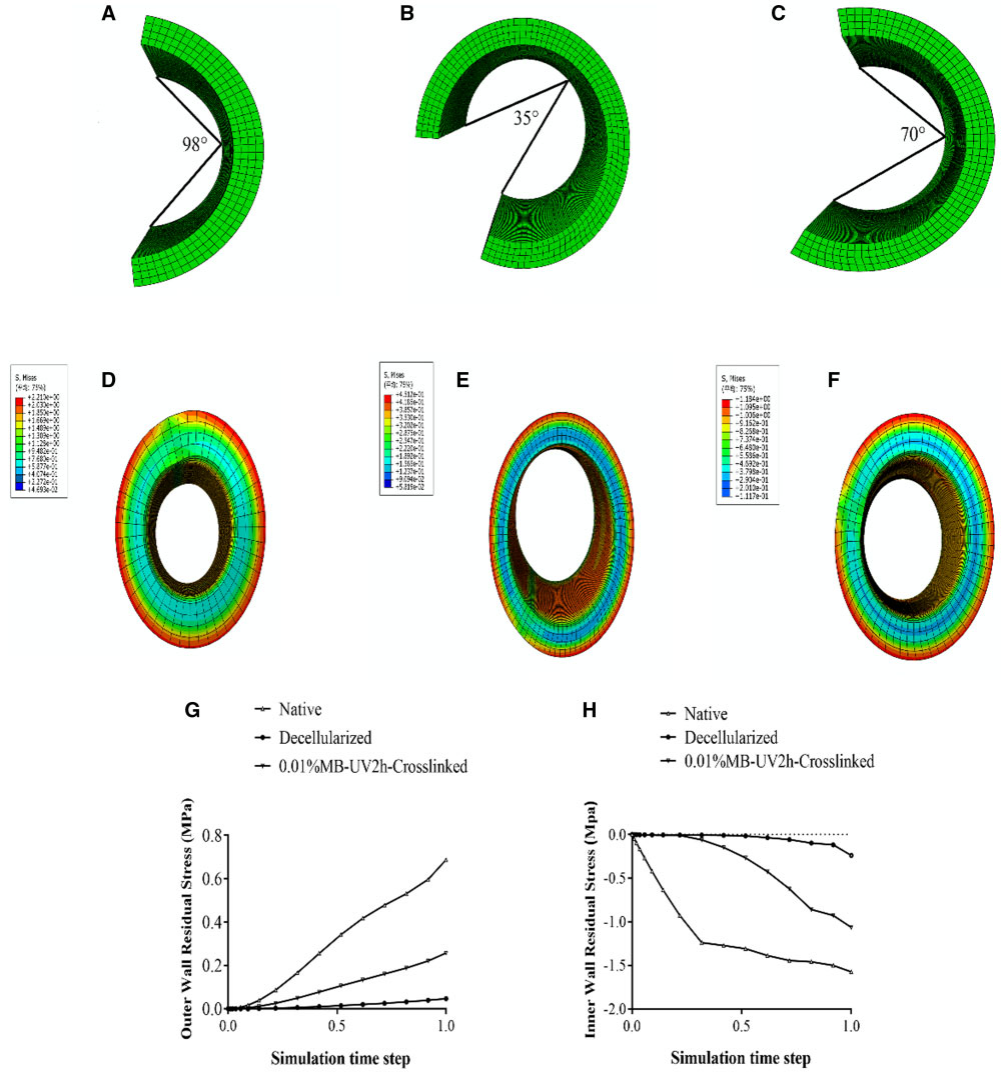
Results of finite element numerical simulation for residual stress. (**A**), (**B**) and (C) represent the average opening angle model of the native, decellularized and 0.01%MB-UV2h-crosslinked arteries. (**D**), (**E**) and (**F**) represent residual stress of the native, decellularized and 0.01%MB-UV2h-crosslinked arteries. (**G**) and (**H**) represent changes in residual stress of outer and inner wall among the native, decellularized and 0.01%MB-UV2h-crosslinked arteries during the whole finite element simulation process

The average opening angles of native, decellularized and 0.01%MB-UV2h-crosslinked arteries, from all the measured data, were 98°, 35° and 70°, respectively (Table 2). The finite element simulation is a process from zero-stress state (open) to no-load state (closed) of the vessel, where the zero-stress state model is shown in Fig. 8A– C. It could be seen that the residual stress distribution of every model gradually decreased from the adventitia to the intima. The maximum residual stress of the 98°model and the 70°model was 0.91 MPa and 0.515 MPa, respectively and the maximum residual stress of the 35°model was 0.193Mpa (Fig. 8D–F). The finite element simulation verified that the residual stress was proportional to the opening angles under the vessel’s elastic state. Residual stress in the adventitia was positive, while that in the intima was negative (Fig. 8G–H). In the simulation process, it could be found that with the progress of time step, the residual stress of 0.01% MB-UV2h-crosslinked arteries was faster and more significant than that of decellularized arteries.

**Table 2.** The average opening angles of native vessels, decellularized vessels and 0.01%MB-UV2h-crosslinked vessels

	Native	Decellularized	0.01%MB-UV2h-crosslinked
Opening angle	98.48°	35.21°	70.67°

## Discussion

At present, most research on the mechanical properties of decellularized arteries have focused on the elastic modulus, burst pressure and suture tolerance [39–44]. Ideal decellularized arteries should have good biomechanical properties and non-immunogenicity while retaining the components of the ECM [45, 46]. However, the process of decellularization increase resulted in an increase in the interval between collagen fibrils, increased fiber mobility and a loss of proteoglycan density. Residual stress on the vessel wall decreased because of changes in the internal structure and the removal of cells. The presence of residual stress is a very favorable condition for blood vessels, to avoid stress concentrations and improve the mechanical properties of the vessels. It is therefore significant for decellularized arteries to reconstruct residual stress. In the present study, we explored the effects of glutaraldehyde crosslinking and photooxidation crosslinking on the opening angle and residual strain of decellularized common carotid arteries in rats, to obtain an effective method for reconstructing the residual stress of the vessel wall.

Histology revealed several distinct aspects of decellularized vessels, which are summarized as follows: (i) extracellular matrix (ECM) was preserved; (ii) the collagen fibers became loose in the adventitia and (iii) there was a separation of elastic fiber layers in the intima (Fig. 2K). The results of the first two points were consistent with previous studies, indicating collagen fibers interactions were altered [11]. The last observation showed that different decellularization methods caused the change in the elastic fiber layers. The process of decellularization determined that the mechanical properties of vessels, especially fiber–fiber mutual effects were changed or broken. The common carotid artery in the rat is twisted from the proximal end to the far side, and the external environment of the nodes is different. Therefore, the opening angles of different sections of a vessel showed irregular changes (Fig. 3A). As is consistent with previous findings [21–24], a decrease of opening angles of decellularized arteries revealed that residual stresses were alleviated. Vascular smooth muscle cells adjusted stress and elastin fibers contribute to residual stress [4, 5]. Increased porosity and the disruption of cell–fiber interactions were caused by the removal of cells which led to the release of residual stresses to decrease opening angles. These internal structural changes also resulted in decreased extensibility and increased stiffness. The increase of tissue stiffness made tissue ductility decreased, which may be related to the decrease of opening angle in decellularized arteries.

The properties of vessel wall greatly affected stress distribution [47, 48]. Residual strain distribution is essential in evaluating residual stress [22]. The outer wall residual strain of decellularized vessels decreased, but the inner wall residual strain exhibited almost no change, indicating that the loose collagen fibers caused the reduction of the outer wall residual strain only. The process of decellularization increased fiber mobility and contributed to the rotation of fibers to move toward the direction of strain [11]. After the vessels were treated with trypsin, the connections between the collagen fibers in the outer wall were destroyed by enzymatic digestion. After decellularization, collagen fibers in the outer wall of decellularized arteries became looser, and the thickness of the collagen fibers decreased after digestion; the resulting external surface of decellularized arteries presents smooth in some locations (Fig. 5D). However, the separation of elastic fiber layers had little influence on the inner wall residual strain because the elastic fibers may be less sensitive than collagen fibers to the decellularization process involving trypsin [11]. The decreased radial residual strain indicated that the fibers extend both laterally and longitudinally after digestion. The changes in opening angles and residual strain indicated that the structural stability of the vessel was broken by the high fiber mobility and the disruption of cell–fiber interactions, which caused residual stress to decrease. A reduced residual stress in the vessel is problematic in the field of tissue engineering; the obtained decellularized arteries become hard, which is not suitable for revascularization. After the implantation of decellularized arteries *in vivo*, abnormal stress distributions in the decellularized arteries’ walls may aggravate the mechanical property mismatch between the artificial vessels and native vessels due to the stress concentration on the intracavity surface. The endothelial cells implanted on the surface may be in an abnormal state, which can even result in apoptosis or rupture.

In crosslinking experiments, traditional crosslinking reagents such as glutaraldehyde and genipin enhanced the mechanical properties of blood vessels, but they had some defects such as potent cytotoxicity and poor stability [49, 50]. In recent years, many researchers have explored new crosslinking methods. All the different crosslinking techniques were able to improve the biomechanical properties of the material and reduce fineness cytotoxicity [32, 51, 52]. However, none of these studies explored the reconstruction of residual stress.

Glutaraldehyde is the most commonly accepted crosslinking reagent, which has been widely used for biomedical applications since the late 1960s [2]. Glutaraldehyde crosslinking can change the mechanical properties of the vessels, in particular, it can increase the fiber hardness, resistance to fiber rotation and tensile strength and flexibility of the structure, whilst it can reduce the ductility of the artery [1, 10]. In our study, the opening angles of the glutaraldehyde crosslinked arteries were smaller than that of the decellularized arteries. There may be two reasons for this observed phenomenon. The reaction between glutaraldehyde and elastic fibers which had the most significant effect on the residual stress of the vessel wall occurred after crosslinking with glutaraldehyde, which resulted in lower toughness of the vessels and a reduction of opening angles. Secondly, a pull was generated by the reaction between glutaraldehyde and collagen fibers encouraging vasoconstriction.

The arteries were treated with photooxidation crosslinking, different concentrations of methylene blue, and different UV irradiation times were used. The effect for 0.01% of MB with UV irradiation for 2 h was the best among three methods, which resulted in opening angles that were significantly different from that of the decellularized arteries. The reason for this phenomenon may be that the method of 0.005% of MB with UV irradiation for 1 h had little time to illuminate and low concentration. The crosslinking method of 0.01% of MB with UV irradiation for 2 h reached saturation in promoting fibers bonding. When the methylene blue concentration increased to 0.03%, and the UV exposure to 4 h, the opening angles were less than of that of 0.01%MB-UV2h-crosslinked arteries. The common carotid artery’s shape is distorted, resulting in different blood pressure on each vessel segment. Therefore, after the decellularization treatment, the vessel opening angle showed a considerable reduction in the 0–20% segment and a slightly smaller reduction in the 40–100% segment. At this time, the 40–100% segment and the 0–20% segment were very different. After photooxidation crosslinking, the opening angle of each segment in the blood vessel was restored. However, due to the distorted shape of blood vessels and different blood pressure, the 40–100% and 0–20% sections are different. We speculate that photooxidation crosslinking increased the opening angles because the connections between the elastic fiber layers and the collagen fiber layers increased, and the porosity was reduced. Vascular smooth muscle cells adjust stress [4] and elastin fibers [5] contribute to residual stresses. Additionally, loss of proteoglycan density led to a reduction in opening angles in decellularized vessels [11], while proteoglycan located in collagen fibers.

After the optimal crosslinking method was determined, the decellularized arteries were crosslinked with 0.01% methylene blue + UV irradiation for 2 h. The subsequent mechanical observation of the crosslinked arteries revealed that outer wall residual strain of the vessel increased after crosslinking, and that increase of residual strain mainly came from the contribution of the increase in outer wall strain. TEM revealed that 0.01%MB-UV2h-crosslinked arteries had denser areas of collagen fibers compared with decellularized arteries and that the spacing between fibers seemed to increase. After crosslinking, the radial residual strain did not change significantly, indicating that the crosslinked fiber mainly became laterally tight and did not change much in the longitudinal direction.

This investigation was limited by the use of rat carotid arteries and a particular decellularization treatment. The decellularization methods based on trypsin-digestion were used in many researches and based on this, it is meaningful to obtain a method to increase both the residual stress and the opening angles of the artery. As a core protein attached to proteoglycans, glycosaminoglycan (GAG) is an essential component of ECM composition, growth, and remodeling, especially in the steady-state process. The reduction of GAG increased fiber spacing and mobility of vascular tissue, leading to increased stiffness. Previous studies have found that GAG decreases residual stress affecting blood vessels and leads to a decrease in the opening angle of decellularized arteries. Photooxidation crosslinking increases the opening angle of decellularized arteries to recover residual stress, and GAG may also be affected by photooxidation crosslinking. The next step is to explore the micro-mechanism of photooxidation crosslinking to recover opening angle, especially the change of GAG.

## Conclusion

By exploring different crosslinking methods, we have found that the photooxidation crosslinking could restore the residual stress of the decellularized arteries. The connections between the collagen fibers and elastic fibers increased with the fiber density increasing. In addition, the outer wall residual strain had a significant influence on the circumferential residual strain. Our first finding that the residual stress of decellularized arteries could be recovered by photooxidation crosslinking will provide biomechanical support for the development of new biomaterials of vascular grafts.

## Author contributions

Z.Y., G.W. and L.K. conceived and designed the experiments. J.W. and J.X. performed the experiments. A.G., X.P. and N.K. helped to collect, analyze and interpret the data. J.W. and X.M. wrote the draft of manuscript. G.W., Z.Y. and C.D. revised the manuscript. N.W., R.H. and H.Z. participated in discussion and improvement of the manuscript. All authors agree to be accountable for all aspects of the work. All authors gave final approval for publication and agree to be held accountable for the work performed therein.

## Funding

This work was supported by grants from the National Key R&D Program (2016YFC1101101, 2016YFC1102305), Preface Technology Project of Chongqing (cstc2018jcyjAX0530, cstc2019jcyj-msxmX0329), Fundamental Research Funds for Central Universities (2018CDXYSW0023) and the National Natural Science Foundation of China (30570453, 31971242). We are also thankful for the support from the Chongqing Engineering Laboratory in Vascular Implants, the Public Experiment Center of State Bioindustrial Base (Chongqing), China.

## Ethics

All animal protocols used in this study were approved by the Ethical Committee of Chongqing University and complied with the State Scientific and Technological Commission of Animal management regulations (SSTC Publication Chapter 4, revised 2007).

**Conflict of interest statement**: The authors declare that they have no conflict of interest.
